# A study exploring predictors of cell phone use while walking among adolescents based on theory of planned behavior

**DOI:** 10.1186/s12889-025-24047-7

**Published:** 2025-08-22

**Authors:** Biaoqian Tang, Jun Ren, Yiyang Li, Koustuv Dalal, Fenfen Li, Xiaoya Yin, Bohao Chen, Wenbiao Zhao, Shumei Wang

**Affiliations:** 1 School of Public Health, Fudan University, Shanghai, 200032 China; 2 Department of Chronic Non-Communicable Disease Control and Prevention, Shanghai Changning District Center for Disease Control and Prevention, Shanghai, 200051 China; 3 Department of Statistics and Information, Shanghai Jiao Tong University Medical School Affiliated Ruijin Hospital, Shanghai, 200025 China; 4 Shanghai Fifth People’s Hospital, Shanghai, 200240 China; 5 Division of Public Health Science, School of Health Sciences, Mid Sweden University, Sundsvall, Sweden; 6 Shanghai Songjiang District Central Hospital, Shanghai, 201600 China

**Keywords:** Pedestrian, Theory of planned behavior, Adolescent, Mobile phone, Structural equation model

## Abstract

**Background:**

Walking is a complex activity that requires high levels of perception and cognitive abilities. Healthy pedestrians who use mobile phones while walking will have their decision-making process affected to varying degrees and may be at greater risk of injury. Previous studies have shown that using mobile phones while walking is becoming increasingly common. Therefore, the objectives of this study are to solve the following problems: What factors influence the intention and behavior of teenagers to use mobile phones while walking? Do different factors play the same role in students of different genders, school types, mobile phone dependence, or mental health status? Based on the above results, what measures should we take to reduce the behaviors of teenagers using mobile phones while walking?

**Method:**

This study used a cross-sectional online survey design. The study was conducted in six junior high schools and four senior high schools in Shanghai, China, from December 2019 to January 2020 (*N* = 4,082 students in Shanghai). The questionnaire was designed based on the theory of planned behavior and analyzed by structural equation model analysis and multi-group invariance analysis.

**Results:**

Girls, junior middle school students, and students without mental health problems or mobile phone dependence can better understand the hazards of using mobile phones while walking. They are more willing to accept other people’s suggestions about not using mobile phones while walking. Additionally, they exhibit better self-control and lower levels of intention and behavior when using mobile phones while walking.

**Conclusions:**

The key points to preventing teenagers from using mobile phones while walking are to instill a correct attitude toward dangerous behavior, build a stronger sense of norm, help them form good habits in mobile phone use, and improve their ability to control their behavior.

## Introduction

Walking is a complex activity that requires high levels of perception and cognitive abilities. Even in normal conditions, for healthy pedestrians who can effectively integrate the required information, their decision-making process will be affected to varying degrees if their attention is distracted. Pedestrians may overlook important auditory or visual information. They may make wrong judgments about speed, especially when multiple lanes or vehicles are involved. They may make incorrect judgments about the driver’s intentions and misjudge their ability to pass through the gaps [1–3]. Therefore, pedestrians with inattention may face a greater risk of injury.

In the past 20 years, mobile phone penetration has risen sharply. According to the monitoring data of the Ministry of Industry and Information Technology of China [4], as of July 2019, China had 1.59 billion mobile phone users, with an average of 1.12 mobile phones per person, ranking first in the world. The proportion of junior and senior middle school students owning their own mobile phones reached 71.3% and 86.9%, respectively [5]. Although the development of communication technology has made our lives more efficient and convenient, it has also brought some worrying public health issues, such as using mobile phones while walking. The US National Highway Traffic Safety Administration report [6] showed that 6,283 pedestrians died in traffic accidents in 2018, the highest number of deaths since 1900. Experts have analyzed the distraction caused by using electronic devices as a significant factor contributing to the rapid increase in pedestrian traffic injuries and deaths. The results of our other case-crossover design study also showed that people who use mobile phones while walking are three times more likely to experience road injuries than those who do not (OR = 3.00, 95% CI: 2.04–4.42). Some studies suggest that the dual-tasking of using a mobile phone while walking may be related to cognitive knowledge, such as the findings of Krasovsky et al., which found that better cognitive flexibility was associated with lower gait dual-task costs when texting while walking [7], and that reading while walking with smart glasses resulted in significant additional cognitive load compared to reading on a mobile phone [8].

Several studies have shown that using mobile phones while walking is becoming increasingly common. An observational study conducted by Safe Kids Worldwide on junior and senior middle school students in Beijing, Shanghai, and Guangdong showed that 35.08% of students always or often make calls while walking [9].

On January 14, 2019, the traffic police in Wenzhou City, China, issued the country’s first ticket for pedestrians who were using their phones while crossing the road. Some other countries and regions (such as the Netherlands and Hawaii) also charge fines to persons for distracted walking. Some studies on the effects of the ban on mobile phone use while driving have shown that the ban is difficult to enforce and may have unintended effects, such as tempting people to use hands-free devices [10, 11]. Moreover, walking situations are very diverse, and crossing the road is only a tiny part of pedestrians’ travel situations. Therefore, we tend to use publicity and other interventions to increase pedestrians’ awareness of and understanding of the hazards associated with using mobile phones while walking. This may change their unsafe behavior and achieve more positive and lasting results.

Although observational research can reveal a great deal of information about actual behavior, it cannot clarify the psychosocial factors that may affect behaviors—using a mobile phone while walking is an autonomous behavior. Psychosocial factors help us understand the motivations that lead to such dangerous behavior and can provide interventions to reduce it.

The Theory of Planned Behavior (TPB) is a classic and widely used psychological theory in health science. It explains the general decision-making process of individual behavior from the perspective of information processing, starting with the expected value theory. It assumes that before performing the behaviors, the individuals systematically use and evaluate the available information related to the outcomes of the volitional behaviors. Furthermore, someone’s willingness or intention to behave is the best predictor of these volitional behaviors. TPB can be used to explore the predictors and influence paths of certain behaviors and has been successfully used to predict health-related behaviors, such as nutrition-related behaviors [12], smoking [13], alcohol consumption [14], oral hygiene behaviors [15], and physical activity [16]. Moreover, studies have attempted to apply the TPB to understand distracted driving and walking, as well as the factors that lead people to use mobile phones [17–21]. Koh and Mackert employed a modified Theory of Planned Behavior (TPB) to investigate the predictive factors influencing college students’ texting while walking and examined the differences between sending and receiving text messages [18]. The results showed that subjective norms, personal norms, and self-efficacy were significant predictors of willingness to send and read text messages while walking. A study in Beijing, China [21] showed that perceived risk significantly impacted Beijing drivers’ choice to call or text. However, other studies have shown that perceptions of control and perceived risk are not predictors for making phone calls [19] or texting [17] while driving. The current research focuses on the drivers’ calling and texting behavior, and only a few studies have assessed the behavior of using mobile phones while walking. Additionally, there are significant differences in the research results across various countries and populations. Therefore, it is meaningful to explore the predictive factors that influence Chinese teenagers’ use of mobile phones while walking, and we can use this information to develop targeted interventions.

In this study, questionnaires were designed based on TPB and using structural equation modeling and multi-group invariance analysis. The objective is to solve the following main research problems. What factors influence the intention and behavior of teenagers to use mobile phones while walking? Do different factors play the same role in students of different genders, school types, mobile phone dependence, or mental health status? Based on the above results, what measures should we take to reduce the behaviors of teenagers using mobile phones while walking?

## Methods

### Study design and participants

This study used a cross-sectional online survey design. The study was conducted in six junior high schools and four senior high schools in Shanghai, China, from December 2019 to January 2020. All pre-first-year and first-year students in junior high schools, as well as first-year students in senior high schools, were selected as the research subjects. The study was conducted after the school informed parents and students voluntarily filled out a self-administered questionnaire with informed consent. There were 4392 students who met the inclusion requirements, and 4082 valid questionnaires were collected. The 4,082 participants were 2,179 boys and 1,903 girls, 2,362 junior high school students, and 1,720 senior high school students. The average age of students in the pre-first year of junior high school was 11.36, the first year of junior high school was 12.39, the first year of senior high school was 15.50, and the average age of all students was 13.40. Also, 1,042 students were positive for mental health problems and 997 for mobile phone dependence.

### Measures

The theory of planned behavior is widely used in literature to understand behaviors [22]. There are three main variables in the Theory of Planned Behavior (TPB): attitude toward the behavior, subjective norms (comprising normative beliefs and motivation to comply), and perceived behavioral control. Generally, the better a person’s attitude and subjective norms, the greater the perceived behavioral control and the stronger their intention to perform that behavior. Ultimately, if individuals possess sufficient control, they will execute their intentions when the opportunity arises. Therefore, intention is considered to be the immediate premise of behavior. However, since the executive difficulties exhibited by many behaviors may limit volitional control, it is necessary to consider perceived behavioral control in addition to intention. Perceived behavioral control can represent actual control ability to some extent, which can help predict behavior in practice. Therefore, the Theory of Planned Behavior (TPB) was used in this study to explore the predictors and influence paths of using mobile phones while walking.

The TPB questionnaire was composed of six dimensions with 17 items (see Table 1 for dimensions and items). Each dimension was checked for inter-item correlations, item contribution to scaling reliability and internal consistency, and the extent to which the distributions approximated normality (Table 1). The initial scale reliability for the subjective norm was lower than 0.6The items loading less than 0.6 in the dimension were excluded to improve reliability. All items were assessed with 7-point scales anchored from 1 to 7.

**Table 1:** The planned behavior theory scale for mobile phone use while walking

Dimensions	Item number	Items	Cronbach’s α
Attitude Toward the Behavior	AB1	How serious are the consequences of a traffic accident when using a mobile phone while walking?	0.837
	AB2	To what extent will using a mobile phone while walking affect normal walking?	
	AB3	To what extent will using a mobile phone while walking cause traffic accidents?	
Normative Belief	NB1	Do you think your family agrees that you use your cell phone while walking?	0.901
	NB2	Do you think your friends agree that you use your cell phone while walking?	
	NB3	Do you think your teachers agree that you use your cell phone while walking?	
Motivation to Comply	MC1	How much is you willing to listen to the advice from your family not to use mobile phone while walking?	0.951
	MC2	How much is you willing to listen to the advice from your friends not to use mobile phone while walking?	
	MC3	How much is you willing to listen to the advice from your teachers not to use mobile phone while walking?	
Perceived Behaviour Control	PBC1	Can you control yourself from using your phone when you are bored?	0.847
	PBC2	When you have navigation and other needs, can you control yourself not to use your phone?	
	PBC3	When a call comes in, can you control yourself not to use your phone?	
	PBC4	When a companion who walks with you uses a cell phone, can you control yourself not to use the mobile phone?	
Intention	BI1	Do you have the idea of reducing the use of mobile phones while walking?	0.704
	BI2	How confident are you that you do not use your phone while walking?	
Behavior	B1	How often do you use your phone in different ways while walking?	0.872
	B2	How often do you use mobile phones in different road environments?	

#### Attitude

Three items assessed participants’ attitudes toward using a mobile phone while walking. A score of one to seven ranged from “not significant” to “very significant” or “not serious” to “very serious.” The higher the score, the more likely participants were to recognize the dangers and adverse consequences of using a mobile phone while walking and to hold negative attitudes towards this behavior.

#### Normative Belief (NB)

Three items were used to measure participants’ perception of the behavioral expectations of those who are important to them. A score of one to seven ranged from “strongly disagree” to “strongly agree.” A higher score indicates that the participant is more aware of others’ expectations of not using a mobile phone while walking.

#### Motivation to Comply (MC)

Although in the standard TPB, both normative beliefs and motivation to comply with the subject norm were considered, according to the results of factor analysis, these two dimensions were found to be quite different, so they were subsequently divided into two dimensions in the analysis. To measure the degree of compliance expectations held by those important to them, we used three items. A score of one to seven ranged from “very unwilling to listen” to “very willing to listen.” Higher scores indicate that the participants are more willing to comply with others’ expectations.

#### Perceived behavioral control (PBC)

The extent to which participants perceived they had control over their mobile phone use while walking in different situations was evaluated using four items. A score of one to seven ranged from “out of control” to “very in control.” The higher the score, the greater the perceived behavioral control.

#### Intention

The behavioral intention of using the mobile phone while walking was assessed using two items. A score of 1–7 for item BI1 indicates “no plan,” “tried but not sustainable,” “intentional but not specific,” “attempted and specific,” “tried,” “not used for a week,” and “not used for a month.” A score of 1 to 7 on item BI2 indicates “definitely can not” to “definitely can.” The higher the score, the lower the participant’s intention to use the phone while walking.

#### Behavior

To measure the prevalence of mobile phone use while walking, two items were used. 1–7 mark for “never”, “rarely”, “occasionally”, “sometimes”, “often”, “frequently” and “always”. However, the two items of behavior were reverse-coded so that the effect was in the direction hypothesized by the TPB theory. The higher the score, the lower the frequency of mobile phone use while walking.

#### Mental health status

The mental health status of participants was assessed using the Mental Health Inventory of Middle-school Students (MMHI-60), developed by Wang et al. [23]. This self-report questionnaire consists of 60 items, each rated on a 5-point Likert scale. Higher scores indicated more serious mental health problems. We calculated the mean score of each participant. Moreover, according to the classification standard of MMHI-60, students with an average score of less than two were assigned to the negative mental health group.

In contrast, others were assigned to the positive mental health group. The scale has been widely verified among middle school students in China. The Cronbach’s alpha in this study was 0.987.

#### Mobile phone dependence

The Mobile Phone Addiction Index [24] was used to evaluate the mobile phone dependence of students. This is a self-report questionnaire comprising 17 items, each rated on a 5-point Likert scale. Higher scores indicated higher levels of mobile phone addiction. We calculated the total score of each participant, using three-quarters (45 points) as the cut-off value, and higher than 45 points were defined as positive for mobile phone dependence. The Cronbach’s alpha in this study was 0.955.

### Statistical analysis

#### Structural equation model analysis

Before testing hypotheses, preliminary analyses were conducted. Confirmatory factor analysis was performed to test the structural validity of the TPB scale. Cronbach’s α-test was performed to analyze the reliability of the overall TPB scale and TPB dimensions.

**Fig. 1:**
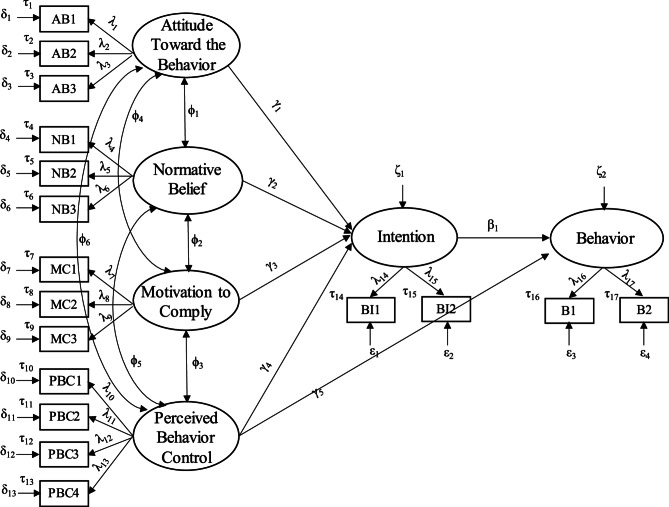
Structural equation modeling of mobile phone use behavior while walking. Legend: λ measurement weight (loading); γ and b structural weight (path coefficient); δ and ε measurement residual; ζ structural residual; ϕ factor covariance; τ intercept (mean score of the item); ▭ observed variable; ⬭ latent variable

The structural equation model (SEM) was conducted to test the research hypothesis using Amos 25.0. The hypothetical model is illustrated in Fig. 1. This hypothetical model is obtained by combining measurement models (MMs) and structural models (SMs) and utilizing several parameters, where squares represent observed variables (original study measurements) and their corresponding unobserved constructs (latent variables in SEMs), depicted as ellipsoids. The arrows from ellipsoids to squares indicate the loading of the observed variables (measurement weights in SEMs), while arrows connecting different ellipsoids illustrate causal paths (structural weights in SEMs).

To analyze the mediating effect of intention between perceived behavior control and behavior, a bootstrap method was used to obtain bias-corrected 95% confidence intervals based on 2,000 bootstrap re-samples.

### Multi-group invariance analysis

The method of multi-group invariance analysis was used to check whether the model had achieved measurement equivalence across different sexes, school types, mobile phone dependence, and mental health statuses and to identify the sources of between-group differences that were meaningful to different groups. First, a baseline model (Unconstrained Model, Model 0) was constructed to determine whether the model should be adopted for each subgroup. If the baseline model for each group is not the same, then none of the more restrictive models in the hierarchy will be able to do so. Next, a total of six invariance tests were performed: (1)

- Model 1: Factorial invariance (null hypothesis: λ_ij_ = λ_ij_); (2)
- Model 2: Intercept invariance (null hypothesis: τ_ij_ = τ_ij_); (3)
- Model 3: Structural path coefficient invariance (null hypothesis: γ_ij_ = γ_ij_ and β_ij_ = β_ij_); (4)
- Model 4: Factor covariance invariance (null hypothesis: ϕ_ij_ = ϕ_ij_); (5)
- Model 5: Structural residual invariance (null hypothesis: ζ_ij_ = ζ_ij_); (6)
- Model 6: Measurement residual invariance (null hypothesis: δ_ij_ = δ_ij_ and ε_ij_ = ε_ij_). The equivalence of factor loadings across groups was tested first because the equivalence of factor loadings is the minimal condition for “factorial invariance” [25]. If the hypothesis of equivalent item-factor loadings is rejected, conduct construct and item-level tests and find solutions. If the hypothesis of equal item-factor loadings is not rejected, we move on to the other invariance tests. Tests of invariance across multiple groups involved a hierarchical ordering of nested models. When models are nested, the difference between the two models can be tested by χ^2^-test. The χ^2^-test is very powerful but sensitive to sample size. Therefore, if the χ^2^ difference was significant (*p*-value < 0.05), the subjective fit indexes (including Tucker Lewis Index (TLI) and Comparative Fit Index (CFI)) were examined to see how much they decline as invariance constraints are imposed. Small decreases in the subjective fit indexes (ΔCFI ≤ 0.01; ΔTLI ≤ 0.05) would suggest that the differences in factor loadings or structural weights are not substantial and are unlikely to affect the interpretation of research results [26]. Mean and standard error of the mean (SEM) were used to describe the score of each item. Independent-sample t-tests were performed to compare each item’s scores between different subgroups.

Statistical analyses were performed using the software Amos 25.0 and IBM-SPSS (version 23.0)

## Results

### Model fit

The hypothetical model demonstrated a good fit (Table 2), indicating that pedestrians’ attitudes, normative beliefs, motivation to comply, and perceived behavioral control had predictive effects on using mobile phones while walking, which were mediated by intention. The model explained 48.7% of the variance in intention and 27.9% of the variance in behavior.

**Table 2:** Evaluation of goodness of fit of the structural equation modeling

Index	Model value	Recommended value	Acceptance
χ^2^/df	9.260,χ^2^ = 972.348, df = 105	< 3 good fit, < 5 reasonable fit, < 8 acceptable fit	no
GFI	0.972	> 0.9	good
CFI	0.981	> 0.9	good
RMSEA	0.045	< 0.05 good fit, < 0.1 reasonable fit	good
SRMR	0.0465	< 0.05 good fit, < 0.1 reasonable fit	good
NFI	0.978	> 0.9	good
TLI	0.975	> 0.9	good

*Abbreviation*: *GFI* Goodness of Fit Index, *CFI* Bentler’s Comparative Fit Index, *RMSEA* Steiger & Lind Root Mean-Square Error of Approximation, *SRMR * Standardized Root Mean-Square, *NFI* Bentler-Bonett Normed Fit Index, *TLI* Tucker Lewis Index

#### Structural model analysis

**Fig. 2:**
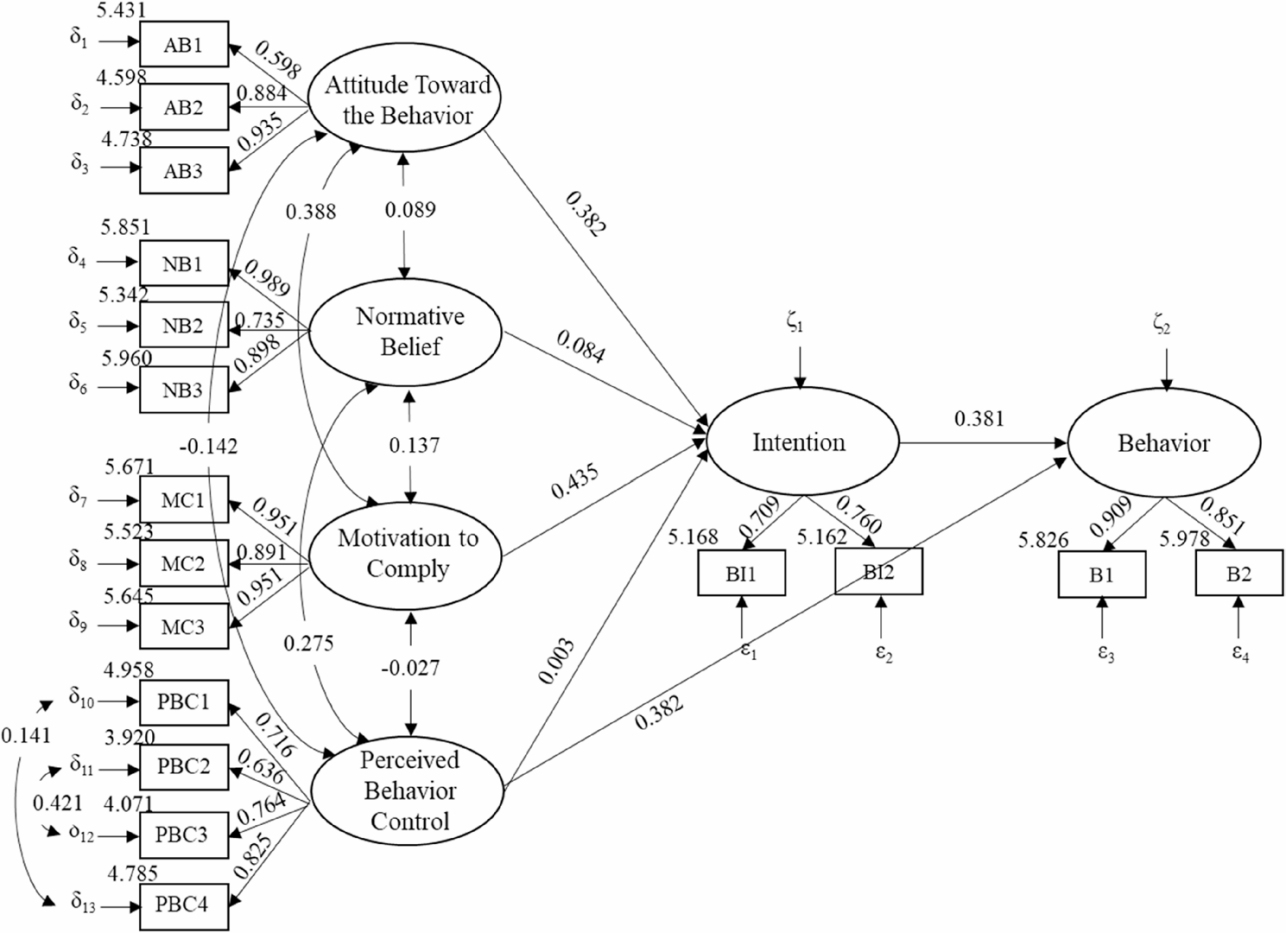
Standardized measurement/structural weights in SEMs

The findings on SMs are presented in Fig. 2. For the following statistics, β reflects a standardized regression (including measurement and structural) weight. Attitude (β = 0.38, *P* < 0.001), normative belief, and motivation to comply (β = 0.44, *P* < 0.001) had significant effects on the intention of using a mobile phone while walking. Intention had a significant and positive correlation with behavior (β = 0.38, *P* < 0.001), and suppressing unsafe intentions can effectively reduce the use of mobile phones while walking.

There was a direct relationship between perceived behavior control and behavior (direct effect = 0.25, *P* < 0.001, 95% CI: [0.22, 0.29]), and the improvement of self-control helped reduce the occurrence of using a mobile phone while walking. Perceived behavior control did not affect behavior through the mediation of intention.

### Measurement model analysis

In terms of attitude, participants generally believed that traffic injuries caused by mobile phones would cause serious consequences (mean = 5.43, std = 0.03). However, they did not think using mobile phones would greatly affect normal walking (mean = 4.60, std = 0.02) or cause traffic injuries (mean = 4.74, std = 0.03). The attitude towards the consequences of the traffic injury had the lowest loading (β = 0.60). In terms of normative belief, students were more likely to comply with the behavior expectations of their family members and teachers (family members: mean = 5.85, std = 0.03, β = 0.99; teachers: mean = 5.96, std = 0.03, β = 0.90). In terms of motivation to comply, students were more willing to follow the advice of their family members and teachers(family members: mean = 5.67, std = 0.03; teachers: mean = 5.65, std = 0.03), the two indicators had the highest load(β = 0.95), and the results were consistent with normative belief. “Feeling bored on the way” was the scenario in which students were most able to control themselves from using mobile phones (mean = 4.96, std = 0.03), followed by “people walking with them using their phones” (mean = 4.79, std = 0.03). The latter was the indicator with the highest loading (β = 0.83). “Having navigation and other requirements” was the scenario in which students could least control themselves not to use mobile phones (mean = 3.92, std = 0.04), followed by “having an incoming call” (mean = 4.07, std = 0.03).

### Multi-group invariance analyses

The χ^2^ difference test between Model 0 and Model 1 was significant for school type, mental health status, and mobile phone dependence. The examination of the subjective fit indexes (ΔCFI and ΔTLI) indicated that such a change reflected largely unsubstantial differences. The parameter estimates of the unconstrained model are presented in Table 3. A series of subsequent nested models revealed that the intercept (Model 2 vs. Model 1) was primarily responsible for the decrements in model fit for school type, mental health status, and mobile phone dependence. Therefore, the item scores of each subgroup were compared.

**Table 3:** Multi-group analysis unconstrained models fit results

Group	χ^2^	df	χ^2^/df	CFI	TLI	RMSEA
Sex	1112.007	210	5.295	0.980	0.974	0.033
School type	1087.045	210	5.176	0.981	0.975	0.032
Mental health	1075.977	210	5.124	0.980	0.974	0.032
Cell phone dependence	1050.795	210	5.004	0.980	0.975	0.031

The invariance analysis results are shown in Table 4. First, multi-group analysis with unconstrained models (Model 0) suggested that the model fit for each subgroup in sex (χ^2^/df = 5.30, CFI = 0.980, TLI = 0.974, RMSEA = 0.033), school type (χ^2^/df = 5.18, CFI = 0.981, TLI = 0.975, RMSEA = 0.032), mental health status (χ^2^/df = 5.12, CFI = 0.980, TLI = 0.974, RMSEA = 0.032), and mobile phone dependence (χ^2^/df = 5.00, CFI = 0.980, TLI = 0.975, RMSEA = 0.031) were good enough, providing evidence of the configural invariance of the construct. Then, to test the invariance of the factor loadings across sex, school type, mental health status, and mobile phone dependence, factor loadings were constrained to be equal across the two groups. Multi-group analysis revealed that this constrained model was acceptable (Table 4). For sex, the χ^2^ difference test between the baseline Model 0 and the constraint Model 1 was not significant.

**Table 4:** Multi-group analysis nested model test results

Group	Nested Model	Δχ^2^	Δdf	*P*	ΔCFI	ΔTLI
Sex	Model 0 vs. Model 1	18.999	11	0.061	0.000	0.001
	Model 1 vs. Model 2	101.671	17	< 0.001	−0.002	0.000
	Model 2 vs. Model 3	40.305	6	< 0.001	−0.001	−0.001
	Model 3 vs. Model 4	113.064	10	< 0.001	−0.002	−0.001
	Model 4 vs. Model 5	145.917	2	< 0.001	−0.003	−0.003
	Model 5 vs. Model 6	423.480	19	< 0.001	−0.007	−0.007
School type	Model 0 vs. Model 1	20.592	11	0.038	0.000	0.001
	Model 1 vs. Model 2	180.742	17	< 0.001	−0.004	−0.002
	Model 2 vs. Model 3	54.330	6	< 0.001	−0.001	−0.001
	Model 3 vs. Model 4	47.368	10	< 0.001	−0.001	0.000
	Model 4 vs. Model 5	0.097	2	0.953	0.000	0.001
	Model 5 vs. Model 6	139.034	19	< 0.001	−0.001	−0.001
Mental health	Model 0 vs. Model 1	47.059	11	< 0.001	−0.001	0.000
	Model 1 vs. Model 2	701.805	17	< 0.001	−0.016	−0.016
	Model 2 vs. Model 3	233.075	6	< 0.001	−0.005	−0.005
	Model 3 vs. Model 4	41.676	10	< 0.001	−0.001	0.001
	Model 4 vs. Model 5	384.755	2	< 0.001	−0.008	−0.009
	Model 5 vs. Model 6	411.491	19	< 0.001	−0.005	−0.005
Cell phone dependence	Model 0 vs. Model 1	68.512	11	< 0.001	−0.001	−0.001
	Model 1 vs. Model 2	768.643	17	< 0.001	−0.017	−0.018
	Model 2 vs. Model 3	306.104	6	< 0.001	−0.007	−0.006
	Model 3 vs. Model 4	64.670	10	< 0.001	−0.002	0.000
	Model 4 vs. Model 5	674.862	2	< 0.001	−0.015	−0.016
	Model 5 vs. Model 6	490.140	19	< 0.001	−0.006	−0.006

**Fig. 3:**
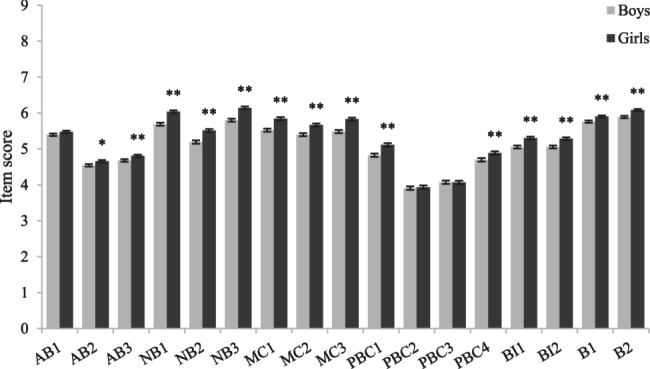
Comparison of item scores between different subgroups in sex. Legend: Bars represent the mean ± standard error of the mean (SEM) of each item score in each subgroup. Independent-sample t tests were performed to compare each item scores between different subgroups. *P<0.05.**P<0.01 (two-tailed test)

**Fig. 4:**
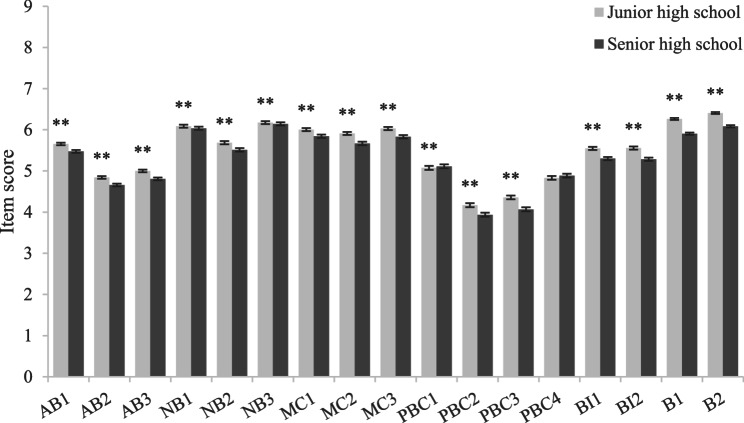
Comparison of item scores between different subgroups in school type. Legend: Bars represent the mean ± standard error of the mean (SEM) of each item score in each subgroup. Independent-sample t tests were performed to compare each item scores between different subgroups. *P<0.05. **P<0.01 (two-tailed test)

**Fig. 5:**
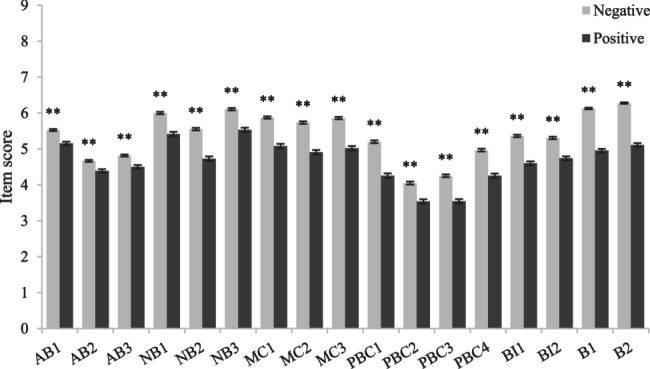
Comparison of item scores between different subgroups in mental health problem. Legend: Bars represent the mean ± standard error of the mean (SEM) of each item score in each subgroup. Independent-sample t tests were performed to compare each item scores between different subgroups. *P<0.05. **P<0.01 (two-tailed test)

**Fig. 6:**
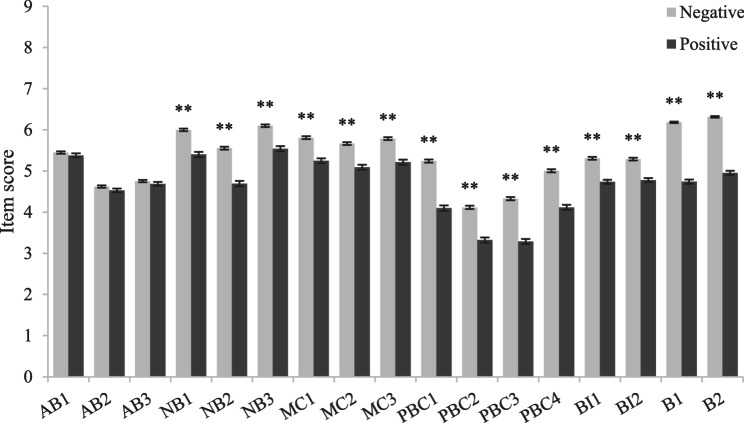
Comparison of item scores between different subgroups in mobile phone dependence. Legend:Bars represent the mean ± standard error of the mean (SEM) of each item score in each subgroup. Independent-sample t tests were performed to compare each item scores between different subgroups. *P<0.05.**P<0.01 (two-tailed test)

Independent-sample t-test results (Figs. 3, 4, 5, 6) showed that among girls (compared to boys), junior high school students (compared to senior high school students), students with no mental health problems (compared to students with mental health problems) and students with no mobile phone dependence problems (compared to students with mobile phone dependence problems) had higher scores in terms of attitude, normative belief, motivation to comply, perceived behavioral control, intention and behavior, indicating that they had better understand the dangers of using mobile phones while walking, were more able to perceive and willing to follow the advice of important people not to use mobile phones while walking, had better control ability, the intention to use mobile phones while walking was lower, and the behavior of using mobile phones while walking was also less.

## Discussion

### TPB had a reasonable explanation for the behavior of mobile phone use while walking

The primary objective of this study was to examine the current usage of mobile phones among young people while walking and to identify the predictors and influencing factors of this behavior. This study examines the differences among students of various genders, school types, mental health statuses, and mobile phone dependence. This research lays the foundation for future research on health interventions aimed at students’ mobile phone problems while walking. To address the initial research problem, we employed a structural equation model to analyze the data. The model showed a good fit, explaining 48.7% of the intentional variance and 27.9% of the behavioral variance. In previous empirical studies based on TPB, the predictive rate of behavioral attitude, subject norm, and perceived behavioral control for behavioral intention remained between 40% and 50%. Meanwhile, behavioral intention and perceived behavioral control contribute 20% ~ 40% to behavioral change [27, 28]. Our model has a good prediction effect.

The results showed that attitude, normative belief, motivation to comply, and perceived behavior control were significant predictors of the use of mobile phones while walking. Girls, junior middle school students, and students without mental health problems or mobile phone dependence can better understand the hazards of using mobile phones while walking. They were more able to perceive and accept other vital persons’ (family members, friends, and teachers) suggestions for not using mobile phones while walking. They had better self-control and lower levels of intention and behavior for using mobile phones while walking.

### Recommendations for targeted intervention strategies based on research results

The study showed that when participants realized that using a mobile phone while walking would seriously affect their normal walking or, to a large extent, cause a traffic accident, they were less likely to use a mobile phone while walking. Therefore, raising students’ mindful awareness of using mobile phones while walking can help students realize that the accident affects not only themselves but also others. Due to the lack of adequate protection for pedestrians, they are more vulnerable to severe injuries in traffic accidents [29]. Existing laws and regulations or traffic safety education emphasize yielding to pedestrians and providing a better travel environment for “vulnerable road users” (including pedestrians, cyclists, and motorcyclists). However, to some extent, this may prompt “vulnerable road users” to show more unsafe traffic behaviors [30]. Judicial data shows that pedestrian accidents account for about 35% of all traffic accidents in China. We must educate motor vehicle drivers to generate public awareness and to use media publicity. On the other hand, we should also pay attention to the education of pedestrians, strengthen their awareness of the dangers of using mobile phones while walking, and vigorously cultivate students’ sense of responsibility as road users. For example, students can be led to watch real accident case data, pictures, or videos in the city by cooperating with traffic police departments or holding related theme presentations or blackboard newspaper design competitions to enhance students’ feelings and awareness.

Subjective norms, especially motivation to comply, had the largest structural weight on intention. This implied that motivation to comply was the most important predictor of intention. This suggested that the expectations of important figures such as teachers, classmates, and parents greatly impacted students. When people around show negative attitudes towards using mobile phones while walking, and students have good feedback on this, students are willing to accept and make changes in their behaviors. Therefore, in future intervention designs, we can fully use the role of teachers, classmates, and parents. Previous studies have found that in terms of dangerous behaviors such as alcoholism, drug use, and texting while driving, peer atmosphere and parental demonstrations all play important roles in changing the students’ behaviors and intentions [31–34]. In future interventions, in addition to the description of the accident case or data, we can also take the form of “psychological team building” to conduct targeted group counseling on students’ subjective norms (including normative belief and motivation to comply), which can be a group with teachers, classmates or parents. Through the sharing and communication of knowledge related to subjective norms, members are guided to form the normative awareness of “not using mobile phones while walking,” consolidate the group, generate group dynamics, and supervise each other through team contracts.

Students with more substantial perceived behavioral control were less likely to use their mobile phones while walking. However, an issue that deserves attention is that the item scores in perceived behavioral control were the lowest. Therefore, it is necessary to improve students’ self-control ability. Professionals should provide students with more easy-to-follow suggestions. It is worth noting that the scores are the lowest when there is a phone call or when they need to use navigation and other functions. In both cases, students find it necessary to use the mobile phone immediately, at which time their self-control awareness is the lowest. Therefore, students should be educated to make complete preparations before going out. They need to be familiar with the travel route, and when using a mobile phone, they should stop walking at a safe place (such as the roadside). At the same time, we should remind students’ parents and companions to avoid making phone calls while the students are walking.

### Item scores vary widely across groups

The analysis of different groups found that for students of various genders, school types, mobile phone dependence, and mental health status, each indicator’s predictive ability was different. The main reason for the discrepancy was that different dimension items had different scores.

In terms of school type, a previous study on making phone calls while driving showed that the driver’s perceived risk awareness increases as age increases [35]. However, this does not seem to apply to our research results on young pedestrians. In this study, senior middle school students had lower attitude scores than junior middle school students. Poysti and Summala’s study found that drivers who think they have better driving abilities are likelier to make phone calls while driving [36]. If this finding applies to pedestrians, another possible reason for the low scores among senior middle school students is that they have more experience and think they can handle complex traffic situations. This attitude leads to a reduction in their awareness of danger. Therefore, in publicity and education, in addition to oral education, we can use scenario simulation experiments and other methods to let students clearly understand the adverse effects of using mobile phones while walking. In addition, senior middle school student’s compliance with the suggestions not to use mobile phones while walking was significantly lower than that of junior middle school students. One possible reason is that high school students are generally in a psychological rebellion period and have low acceptance of suggestions from others [37]. In response to this phenomenon, they may need the guidance of social propaganda and even laws and regulations. Parents can also demonstrate their behaviors, which is part of daily education, instead of just giving orders.

For mobile phone dependence, there was no significant difference in the scores of attitudes between negative and positive students. This result suggests that students who rely heavily on mobile phones can also recognize the hazards of using mobile phones while walking. However, they are more reluctant to follow the advice of others and have more difficulty controlling their behaviors. Some studies suggested that using a mobile phone while walking was just a spatial continuation of the mobile phone dependence [38], which was consistent with our research. Therefore, it is more important for students with mobile phone dependence to change their daily mobile phone habits and strengthen their self-control capabilities.

### Study limitations

This study has several limitations. First, the frequency of mobile phone use was based on students’ self-perceived frequency rather than actual observation results. Different students may have different judgments on frequency, which may lead to deviations in research results. In addition, since this study used the fundamental theory of planned behavior, it did not incorporate the role of social norms in the initial research design. This indicator has significantly affected the intention and behavior of using mobile phones while walking in past studies. Therefore, this study may miss some influencing factors. Finally, this study did not distinguish various ways of mobile phone use, which could have significant differences. However, it used mean values, which could not distinguish and explore these behaviors in-depth. In future studies, different versions of the scale could be developed to compare the differences in predictors of different ways of mobile phone use.

## Conclusions

Despite these limitations, the results have important practical implications. At present, it is typical risky behavior for teenagers to use mobile phones while walking, and there are few related studies. The findings of this study describe the current state of mobile phone use among young people while walking and analyze the psychological pathways that lead to this behavior. The findings can provide technical direction and evidence support for the development of persuasive and targeted intervention programs to prevent the use of mobile phones while walking. The key points to preventing using mobile phones while walking are to initiate teenagers’ correct attitude toward dangerous behavior, build a stronger sense of norm, help them to form good habits in using mobile phones, and improve their behavior control ability. For students of various genders, school types, mental health status, and mobile phone dependence, we should adopt different strategies for different people to improve the effectiveness of the interventions.

## Data Availability

No datasets were generated or analysed during the current study.

## Abbreviations

CFI: Comparative Fit Index
CI: Confidence interval
MC: Motivation to Comply
MMHI: Mental Health Inventory of Middle-school Students
NB: Normative Belief
OR: Odds Ratio
PBC: Perceived behavioral control
SEM: Structural Equation Model
SM: Structural Models
TPB: Theory of Planned Behavior
TLI: Tucker Lewis Index
US: United States
